# A Simple and Sensitive UPLC–UV Method for Simultaneous Determination of Isoniazid, Pyrazinamide, and Rifampicin in Human Plasma and Its Application in Therapeutic Drug Monitoring

**DOI:** 10.3389/fmolb.2022.873311

**Published:** 2022-04-29

**Authors:** Zhimei Jiang, Liang Huang, Lingli Zhang, Qin Yu, Yunzhu Lin, Haotian Fei, Hongxin Shen, Hong Huang

**Affiliations:** ^1^ Department of Pharmacy, Evidence-Based Pharmacy Center, West China Second University Hospital, Sichuan University, Chengdu, China; ^2^ Key Laboratory of Birth Defects and Related Diseases of Women and Children, Ministry of Education, Sichuan University, Chengdu, China; ^3^ Institute of Drug Clinical Trial/GCP Center, West China Second University Hospital, Sichuan University, Chengdu, China

**Keywords:** isoniazid, pyrazinamide, rifampicin, therapeutic drug monitoring, UPLC–UV

## Abstract

As the first-line clinical drugs for tuberculosis (TB), isoniazid (INH), pyrazinamide (PZA), and rifampicin (RMP) are playing important roles for preventing the rapid spread of TB. Precise quantification of these drugs in biological samples is crucial to evaluate or improve the efficacy of advanced TB drug delivery systems, which are designed for reducing drug resistance, minimizing side effects, etc. Herein, a simple and sensitive method based on UPLC–UV was established and investigated for simultaneous quantification of PZA, INH, and RMP in human plasma and was applied to anti-TB drug therapeutic drug monitoring. The analytes were implemented on an HSS T3 C18 column at 40°C. The separation was performed with a gradient elution with methanol–acetonitrile–water (3:3:94) at 0.1 ml/min. The analysis only involved plasma with a small volume of 100 µL and a rapid one-step protein precipitation with methanol–acetonitrile (1:1). The results showed that the calibration curves for INH, PZA, and RMP were linear in a range of 0.5–20 μg/ml, 5–60 μg/ml, and 5–60 μg/ml, respectively. The intra- and inter-day precisions were both smaller than 15%, and the lower limit of quantitation (LLOQ) was identifiable and reproducible at 0.5 μg/ml for INH and 5 μg/ml for both PZA and RMP, respectively. The target drugs in plasma were stable after 21 days of storage at −80°C. The results indicated that our developed method is suitable for the simultaneous monitoring of INH, PZA, and RMP in human plasma.

## Introduction

Tuberculosis (TB) is ranked within the top 10 causes of death in the world, and the leading cause of death from infectious diseases (above HIV and AIDS). In the year of 2019, around 10 million people were confirmed with TB within the world. Meanwhile, drug resistance to TB has become a serious threat to public health. According to the Global Tuberculosis Report, 2020 posted by the WHO, almost half million patients with TB developed drug resistance to rifampicin TB (RR-TB) in 2019, of which 78% of them got multidrug-resistant TB (MDR-TB). The three countries with the largest burden of TB were India (27%), China (14%), and the Russian Federation (8%) (WHO, Global Tuberculosis Report, 2020). Statistically, 3.3% new TB patients and 17.7% TB patients who were previously treated developed MDR or RR-TB.

Pyrazinamide (PZA), isoniazid (INH), and rifampicin (RMP) are the most commonly used drugs for TB therapy in clinics. Particularly, multidrug therapies, for instance, “2S(E)HRZ/4HR (streptomycin or ethambutol, INH, RMP, and PZA were used once a day in the first 2 months of fortification; for next 4 months, continue to use INH and RMP once a day),” are often recommended for TB patients, and the therapeutic period can last for at least 2 months. When the initial treatment regime fails, the therapeutic period can even be longer. Although those regimens are effective for most patients who developed TB, considerable patients show poor responses to the regimens and finally suffer from treatment failure, which could be partly caused by drug resistance (Du Preez and Loots, 2018). Previous studies have indicated that numerous factors could cause the poor response of patients to TB drugs. First, extended drug exposure and an optimum serum concentration level are found to be vital for improving the drug responses. For instance, Prahl et al. observed that lower serum concentration of anti-TB drugs led to treatment failure and drug resistance to TB (Prahl et al., 2014), which is especially the case for patients with concomitant diseases, such as HIV, type 2 diabetes, and gastrointestinal tract diseases (Hemanth Kumar et al., 2017). They expected that the reason could be that these patients with concomitant diseases could easily suffer from a poor drug absorption and drug–drug interaction. On the other hand, excessive TB drug plasma concentrations could cause some side effects, including hepatic problem and thrombocytopenia (Satyaraddi et al., 2014).

To overcome the limitations of traditional TB drugs, various nanoparticle drug delivery systems have been developed and investigated in the past two decades, including polymeric nanoparticles, lipid-based nanoparticles, metal nanoparticles(Costa-Gouveia et al., 2017a). As compared to free TB drugs, the nanoencapsulation could help improve the delivery of drugs to the targeting sites (both in cell and tissue levels) and also decrease the drug administration frequencies by controlling drug delivery kinetics and improving intracellular delivery, resulting in dose reduction and efficacy enhancement at the same time (Shegokar et al., 2011; Gordillo-Galeano et al., 2021). In addition, researchers have found that by co-delivering anti-TB drugs and their “boosters” using nanoparticles, the drug resistance effect can be significantly reduced (Costa-Gouveia et al., 2017b). Precise quantification of anti-TB drugs in biological samples, desirably simultaneous quantification of multiple anti-TB drugs, is crucial for evaluating or improving the efficacy of these modern drug delivery systems.

On the other hand, therapeutic drug monitoring (TDM) has been receiving increasing attention from clinics as TDM can help clinics in adjusting anti-TB drug dosages for patients with low responses (Verbeeck et al., 2016). However, the lack of effective detection methods of TB drugs in plasma samples has significantly limited the wide application of TDM for anti-TB drugs. To determine plasma concentrations of the three first-line anti-TB drugs at the same time, a simple and accurate method is urgently required. Several analytical methods such as HPLC–MS/MS (Kim et al., 2015; Shah et al., 2016; Lei et al., 2019; Wu et al., 2019; Wang et al., 2020; Zheng et al., 20202020), HPLC–UV (Moussa et al., 2002; Allanson et al., 2007; Chen et al., 2010; Mourah et al., 2016; Hernandez-Gonzalez et al., 2021), and HPLC–FLD (El-Yazigi and Raines, 1992) have been reported to quantify these three drugs. Among these, HPLC–MS/MS is the most commonly used method, but it is difficult to widely promote in clinics because of its high costs and complex operation processes. Meanwhile, the reported HPLC–UV or HPLC–FLD methods also have their own disadvantages, such as time-consuming running period, complex sample pretreatment, and lack of internal standard.

In the current study, a simple and reliable UPLC–UV method to simultaneously determine plasma concentration of PZA, INH, and RMP with caffeine as an internal standard was developed, validated, and applied for the three anti-TB drugs. The developed method is simple, efficient, and economy- and environment-friendly, which could effectively promote the routine monitoring of the three anti-TB drugs.

## Materials and Methods

### Chemicals

Isoniazid (Lot: 003zlS, purity: 100.0%) reference was obtained from European Directorate for the Quality of Medicines and HealthCare European Pharmacopoeia (Ph. Eur.). Pyrazinamide (Lot: 100178-201104S, purity: 99.9%), rifampicin (Lot: 130496-201403, purity: 98.8%), and caffeine (Lot: 171215-200608, purity: 100%) references were acquired from the National Institutes for Food and Drug Control (Beijing China). Acetonitrile and methanol were chromatographic-grade and procured from Fisher^®^ (Thermo, America). Ultrapure water was prepared *via* Milli-Q^®^ Direct 8, filtered, and used throughout the experiment. All the other reagents were of analytical grade and used as received.

### Instrumentation and Chromatographic Conditions

The UPLC–UV system included a Waters H-class equipped with a F19QSP315A pump, E19FTP327G automatic injector, and E19TUV823A UV detector (Waters, Shanghai, China). Three anti-TB drugs and the internal standard (IS) were analyzed on an Acquity UPLC HSS T3 C18 column at 40°C (2.1 × 100 mm, 1.8 μm; Waters Corporation, Shanghai, China). The running time for all compounds was 14 min. The detection wavelength for INH, PZA, and IS was set at 268 nm, and the detection wavelength of RMP was set at 340 nm. Mobile phases A and B were methanol–acetonitrile (1:1) and ultrapure water, respectively. A gradient elution was carried out according to the steps in Table 1.

**TABLE 1:** Gradient elution steps in the developed method.

Time/min	Flow rate/mL·min^−1^	A/%	B/%	Detection wavelength/Nm
0–6	0.1	6	94	268
6–9	0.3	30	70	268
9–12	0.3	80	20	340
12–14	0.1	6	94	268

### Preparation of Standard Solutions, Calibration Standard Solutions, and Quality Control Samples

Standard primary solutions of INH, PZA, RMP, and IS were separately dissolved in methanol to prepare stock solutions with a concentration of 1 mg/ml, 2 mg/ml, 2 mg/ml, and 1 mg/ml, respectively. Standard working solutions of INH (10, 50, and 150 μg/ml), PZA (100, 200, and 400 μg/ml), and RMP (100, 200, and 400 μg/ml) were diluted with ultrapure water–methanol (9:1). All of these standard solutions were stored at −20°C and warmed to room temperature. The standard samples used for building calibration curves and the quality control (QC) samples were freshly prepared by spiking different standard working solutions in blank plasma. The calibration curve samples of INH, PZA, and RMP had a concentration range of 0.5–20 μg/ml, 5–60 μg/ml, and 5–60 μg/ml, respectively. QC samples were 1 (LOQ, low of quantitation), 5 (MOQ, medium of quantitation), and 15 (HOQ, high of quantitation) µg/mL for INH; 10 (LOQ), 20 (MOQ), and 40 (HOQ) µg/mL for PZA; and 10 (LOQ), 20 (MOQ), and 40 (HOQ) µg/mL for RMP, respectively.

### Sample Pretreatment

To obtain the blood samples from volunteers, 1 ml blood was collected from venous, after which the serum was obtained by centrifuging the samples at room temperature (5000 rpm, 5 min). Next, the serum sample was transferred into Eppendorf tubes and stored at a temperature of −80°C for further measurements. A protein precipitated method was followed for extraction of INH, PZA, RMP, and IS from the collected plasma. In the following step, 100 µL of the plasma samples were added into 300 µL methanol–acetonitrile (1:1, include IS 10 μg/ml) and mixed for 3 min by vortexing, and the supernatant was collected by centrifuging at 14,000 rpm for 5 min. The supernatant was finally diluted with Milli-Q water 10 times, and 10 µL of the solution was analyzed by the UPLC system.

### Method Validation

The developed UPLC method was validated according to the criteria of industrial guidance for bioanalytical method validation of the FDA, including selectivity, linearity, recovery, precision, accuracy, and stability (FDA, 2018, Guidance for industry, bioanalytical method validation).

#### Selectivity

To investigate possible interference from endogenous components in the samples on the analysis, six different batches of blank human plasma were inspected at the HPLC peak regions of INH, PZA, RMP, and IS.

#### Recovery

The recoveries of the analytes and IS were calculated as the ratio of the peak area of the drug samples spiked after protein precipitation to that of standard solutions at the same concentration. The recoveries were measured by using samples at three concentrations with five replicates for each concentration. The samples were processed with a similar method as mentioned earlier (*Sample Pretreatment Section* sample disposition). Recovery of IS was determined at an IS concentration of 30 μg/ml.

#### Calibration Curves and LLOQ

Calibration curves of INH (0.5, 1, 2.5, 5, 10, 15, and 20 μg/ml), PZA (5, 10, 15, 20, 30, 40, and 60 μg/ml), and RMP (5, 10, 15, 20, 30, 40, and 60 μg/ml) were established in five replicates. The calibration curve was expressed as an equation y = ax + b, where y is the ratio of the peak area of the drugs to that of IS and x is the concentration of drugs. The linearity of the calibration curves was evaluated by linear regression with Excel software. The primary criterion for each back-calculated standard concentration was ±15%, while that of LLOQ was ±20%.

The LLOQ was determined by seeking the lowest concentration of the drugs on the calibration curves that possess satisfactory recovery and repeatability. Additionally, the response of LLOQ should be at least 10 times of that of blank samples. According to the criteria of the FDA, the accepted levels for accuracy and precision of LLQC should not exceed ±20%.

#### Precision and Accuracy

The intra-assay precision and accuracy of the developed analysis method were evaluated by measuring five replicates of INH, PZA, and RMP at three different concentration levels in the plasma of the volunteers. The inter-assay precision was measured by analyzing the responses of the samples with three different concentrations on three consecutive days. The criteria of accuracy and precision were set as within ±15% of the nominal values and ±15% relative standard deviation (RSD), respectively.

#### Stability

The stabilities of the analytes were assessed in five replicates using three different QC samples under different conditions (i.e., stored at 25°C and light-free for 4 h, at −80°C for 1, 3, 7, and 21 days and once freeze–thaw from −80°C to room temperature, and the post-extracted samples stored in the sample tubes at 25°C for 8 h). The concentrations of the samples used for stability studies were determined by using the calibration curve, following with a comparison with fresh samples. The deviations for all the samples from nominal concentrations should be lower than ±15%.

To investigate the stability of the stock solutions, the solutions in triplicate were stored for 30 days at −20°C, and their peak areas were compared with that of the freshly prepared samples.

### Application

All the experimental procedures were approved by the West China Second University Hospital, Sichuan University. Patients were selected for TDM because of poor response and treatment failure or drug resistance. At 2 h after medication administration, venous blood of the patients was collected and separated by centrifuging at 4000 rpm for 5 min. The plasma samples were stored at −80°C for further analysis.

## Results

### Method Validation

#### Selectivity

**FIGURE 1:**
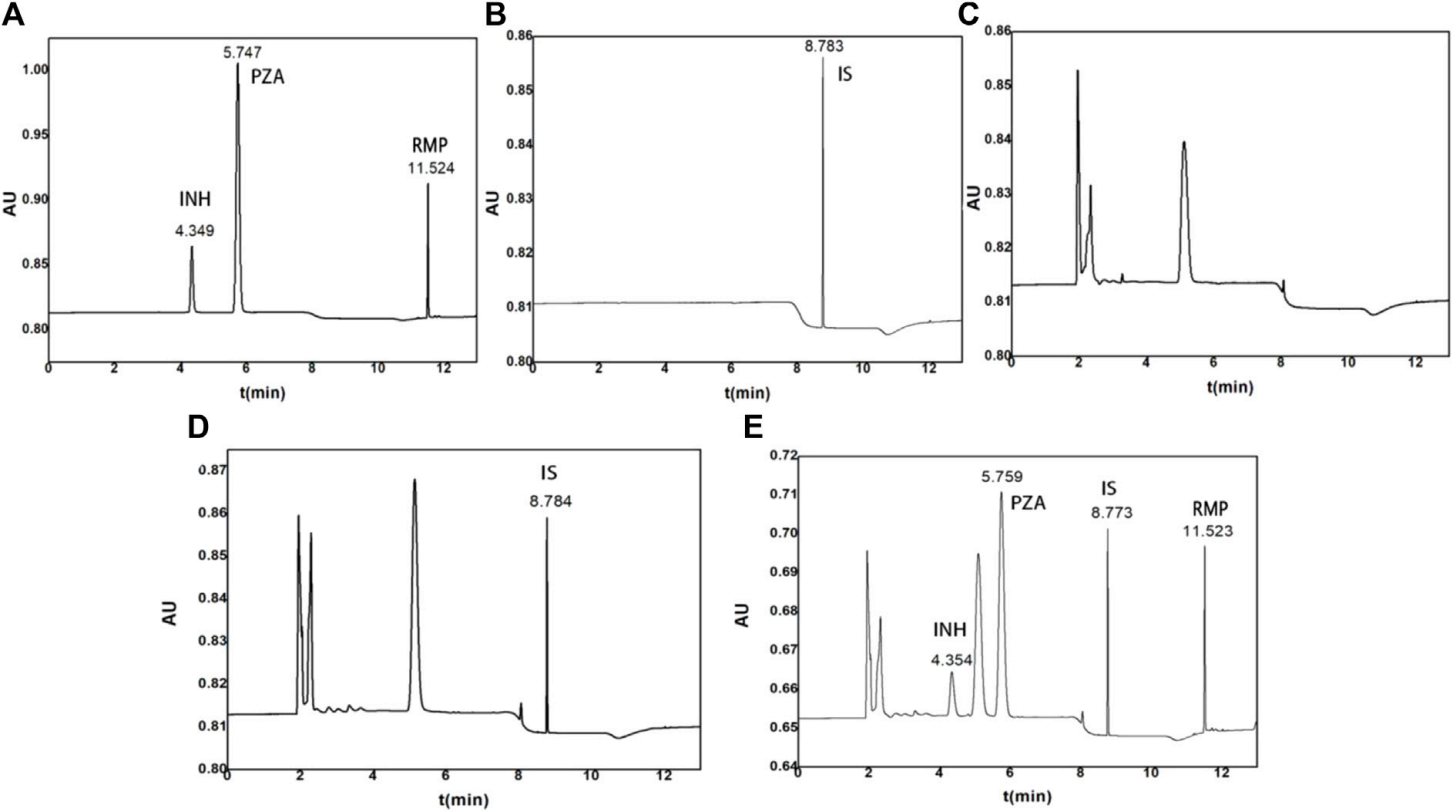
Representative chromatograms of **(A)** standard solutions with INH, PZA, and RMP, **(B)** standard solutions with IS, **(C)** blank control plasma, **(D)** control plasma spiked with 10 μg/ml IS, and **(E)** control plasma spiked with 15 μg/ml INH, 40 μg/ml PZA, and 40 μg/ml RMP.

The retention time of INH, PZA, IS, and RMP was 4.370, 5.788, 8.784, and 11.518 min, respectively (Figure 1). Additionally, the blank human plasma did not show any significant interferences on the retention time of the analytes and IS.

#### Recovery

The recovery percentages of the drugs are presented in Supplementary Table S1. The results showed that all the three drugs had a recovery ranging from 86.61 to 102.05% with little variation at three different QC levels. The recovery of IS was 90.85 ± 2.20%. In sum, our results showed that methanol–acetonitrile (1:1)-mediated protein precipitation resulted in a high and satisfactory extraction recovery.

#### Calibration Curves and LLOQ

The results showed that our method led to a good linearity for all the three analytes. All of the correlation coefficients (*R* ^2^) were over 0.99. The equations of the calibration curves were as follows: *y* = 6.56*10^−5^x-7.04*10^−5^ (*R* ^2^ = 0.9995) for INH, *y* = 1.33*10^−1^x+3.13*10^−2^ (*R* ^2^ = 0.9994) for PZA, and *y* = 1.69*10^−2^x-4.53*10^−2^ (*R* ^2^ = 0.9981) for RMP.

LLOQ was determined in five replicates of spiked plasma sample at a concentration of 0.5 μg/ml for INH, 5 μg/ml for PZA and RMP. The determined LLOQ were (0.532 ± 0.05) µg/ml for INH, (4.868 ± 0.19) µg/ml for PZA, and (4.820 ± 0.12) µg/ml for RMP.

The deviations of all calibration samples were acceptable as the RSD and LLOQ were located within ±15% and ±20%, respectively.

#### Inter- and Intra-Day Precision and Accuracy

The results of inter- and intra-day precision and accuracy results are summarized in Supplementary Table S2. Both inter- and intra-day occasions conformed to the criteria requirements.

#### Stability

The stability of the three analytes in human plasma is shown in Table 2. The data showed that INH, PZA, and RMP were stable under the evaluated conditions, revealing that the processing and analysis did not affect the samples. Specifically, the three analytes in human plasma were not affected after storage at −80°C for 21 days and one freeze–thaw cycle. Additionally, the processed samples were stored in the autosampler vials at room temperature for 8 h prior to analysis. These results showed that this process had no observed effect on the analysis.

**TABLE 2:** Stability of analytes in human plasma (*n* = 5).

	C (μg/mL)	25°C and light-free, 4 h			Post-extracted samples 25°C, 8 h			One freeze–thaw cycles			−80°C, 21 day		
		Mean ± SD	Accuracy%	RSD	Mean ± SD	Accuracy%	RSD	Mean ± SD	Accuracy%	RSD	Mean ± SD	Accuracy%	RSD
INH	1	0.978 ± 0.03	97.78	3.37	0.973 ± 0.02	97.32	1.64	0.905 ± 0.04	90.52	4.04	0.907 ± 0.08	90.68	9.02
	5	5.093 ± 0.03	101.86	0.63	5.026 ± 0.02	100.53	0.45	4.947 ± 0.04	98.94	0.53	5.293 ± 0.03	105.86	0.64
	15	15.229 ± 0.03	101.53	0.19	15.104 ± 0.01	100.70	0.09	14.916 ± 0.04	99.44	7.25	15.368 ± 0.46	102.45	2.98
PZA	10	9.676 ± 0.72	96.76	7.40	9.787 ± 0.02	97.87	0.23	9.701 ± 4.04	97.01	4.71	9.789 ± 0.45	97.89	4.58
	20	20.802 ± 0.07	104.01	0.34	20.480 ± 0.27	102.40	1.32	20.105 ± 0.04	100.53	0.15	20.818 ± 0.02	104.09	0.11
	40	40.579 ± 0.04	101.45	0.11	40.182 ± 0.02	100.46	0.04	39.930 ± 1.04	99.82	2.78	41.783 ± 1.80	104.46	4.30
RMP	10	8.973 ± 0.25	89.73	2.76	9.124 ± 0.26	91.24	2.85	9.298 ± 5.04	92.98	5.70	9.242 ± 0.28	92.42	3.07
	20	19.411 ± 0.58	97.05	2.97	19.674 ± 0.47	98.37	2.37	19.712 ± 2.04	98.56	1.28	19.255 ± 0.29	96.28	1.53
	40	39.978 ± 1.91	99.94	4.77	42.769 ± 0.66	106.92	1.55	41.765 ± 5.04	104.41	3.61	41.981 ± 0.53	104.95	1.27

### Application

Finally, the validated UPLC–UV analysis method was used to determine the concentrations of INH, PZA, and RMP in plasma from four TB children. These children were all diagnosed with tuberculous meningitis, and the therapeutic efficacies were still poor after 10–25 days of treatments. After consultation with clinical pharmacists, the clinician decided to conduct TDM to find out the causes of the poor drug responses. The UPLC–UV determination results are shown in Table 3. As the recommended concentrations of INH, PZA, and RMP are 3–6, 20–50, and 8–24 μg/ml, respectively (Alsultan and Peloquin, 2014; Perumal et al., 2020), the number of patients with actual drug concentrations within the recommended range were 2, 3, and 1, respectively, for INH, PZA, and RMP.

**TABLE 3:** INH, PZA, and RMP TDM outcomes for the four tuberculosis patients.

		A	B	C	D
Age (y)		4.83	5	13.17	7.83
Weight (kg)		17.5	17.2	44.5	35.6
Sex		Male	Male	Male	Female
Drugs and dose		INH 0.2 g, PZA 0.75 g, RMP 0.3 g, and EMB 0.5 g	INH 0.175 g, PZA 0.5625 g, RMP 0.25 g, and EMB 0.3125 g	INH 0.5 g, PZA 1 g, and RMP 0.6 g	INH 0.4 g, PZA 1 g, and RMP 0.5 g
Concentration (μg/ml)	INH	4.161	0.717	19.587	5.436
	PZA	10.490	29.453	38.684	20.372
	RMP	23.324	5.723	8.672	7.735

## Discussion

### Optimization of Chromatographic

To obtain an optimal UPLC separations and peak shapes, several types of columns including a Shim-pack Scepter Diol-HILIC-120 (2.1 × 100 mm, 1.9 μm), Waters BEH C18 (2.1 × 100 mm, 1.7 μm), and Waters HSS T3 (2.1 × 100 mm, 1.8 μm) were tested. INH could not retain on the Shim-pack Scepter Diol-HILIC-120 column, and its retention time was less than 0.5 min. As for BEH C18, it was found that only when the proportion of the water phase was increased to 97%, the separation and peak shapes for all the three analytes and IS were satisfactory. However, the silica gel packing of the BEH C18 phase is easy to be collapsed in high proportion of water. Eventually, the HSS T3 column was chosen, which can withstand 100% water phase.

Several compositions (0.1% trifluoroacetic acid, formic acid, 10 mM potassium dihydrogen phosphate, methanol, ammonium acetate, and acetonitrile) and proportions of the mobile phases were investigated to obtain the optimal separation degree and the shortest running time. Interestingly, it was found that when using the single-organic phase-water (no matter methanol–water or acetonitrile–water), the separation of INH, endogenous impurity, and PZA was not satisfactory. Additionally, if the proportion of methanol was increased and the proportion of acetonitrile was decreased while the total proportion of organic phase was maintained unchanged, INH and impurity cannot be separated. On the contrary, PZA and impurity could not be separated when the percentage of acetonitrile increased and the proportion of methanol decreased. Finally, the influence of pH of the mobile phase on the separation and peak shape was also studied. It was found that the separation and peak shape of these four analytes were not affected by the mobile phase pH (3, 6, or 9).

In most of the previous studies, the temperature of the column was set at 50°C, but high column temperature will shorten the life of the column. Therefore, the influence of column temperature on the retention time and response was also studied. It was found that when the column temperature increased, the retention time of the analytes decreased, while the response increased. After weighing the influence of column temperature on column life, retention time, and response of the analytes, the final column temperature was set at 40°C.

### Sample Pretreatment

Several pretreatment methods were screened to obtain simple and optimal plasma processing, including liquid–liquid extraction (LLE), solid-phase extraction (SPE), and protein precipitation. SPE was excluded due to its disadvantages including time-consuming and high cost. Various extraction solvents were investigated for LLE, including ethyl acetate, dichloromethane, methyltert-butyl ether, petroleum ether, and their mixtures. However, the obtained extraction recovery and purification of the analytes by using these organic solvents were not satisfying. Finally, methanol-acetonitrile (1:1) was selected and used to minimize potential interferences by protein precipitation. We observed that protein precipitation led to high and consistent extraction recovery and showed no interferences for the analytes. As compared with SPE and LLE, protein precipitation was rapid, simple, and economical.

### Novelty of the Developed Method

Compared with the analytical methods reported in previous studies (Moussa et al., 2002; Allanson et al., 2007; Chen et al., 2010; Hernandez-Gonzalez et al., 2021), our developed UPLC–UV method improved retention times, reduced the volume of organic reagent, reduced the sample volume, and achieved symmetric peak shape. The total running time was 14 min, which was much shorter than that of the reported methods. The volume of organic reagent used in this method is about one-tenth of the existing methods, which is environment-friendly and cost-effective. In addition, this method only needs 100 μL of plasma, and therefore can increase the compliance of patients, and is more suitable for the antituberculosis TDM in children. In addition, the one-step protein precipitation method for sample pretreatment in our current study was faster and more efficient than the previously reported two-step liquid–liquid extraction and solid-phase extraction methods. Finally, we successfully applied the developed method to clinical practice for TDM of three of the first-line anti-TB drugs, including INH, PZA, and RMP. Furthermore, our developed method possesses superiorities for pharmacokinetic analysis of anti-TB drugs due to its rapid and simple processing steps, optimal analytical sensitivity, and accuracy.

### TDM Results

The plasma concentration of the three anti-TB drugs can be affected by many factors, such as the combination of other diseases, drugs, and food. Patients who take other drugs at the same time and the interaction between drugs can reduce or delay the absorption of anti-TB drugs. Food can reduce the absorption of isoniazid and rifampicin, especially high-fat diet, which can reduce the absorption of isoniazid by 51%. Although factors such as age, gender, diet, liver function, renal function, basic diseases, and combination of drugs may affect the efficacy and toxicity of the drug, it is currently considered that 20–95% of the individual differences are determined by the individual genetic background.

The INH plasma concentration of patient B was lower than the recommended concentration and that of patient C was much higher than the recommended concentration. The main factors that affect the plasma concentration of INH may be the NAT2 genetic polymorphism. Moreover, the RMP plasma concentrations of patients B, C, and D were lower than the recommending concentration, and the main reason may be the T_max_ delay. We recommend that venous blood samples of these three patients should be collected at 6 h after medication administration.

## Conclusion

A rapid, sensitive, simple, and stable method was successfully developed and applied in TDM. To our knowledge, this is the first study that simultaneously determined INH, PZA, and RMP concentrations in human plasma by UPLC–UV. Compared with HPLC–UV methods, shorter analytical time, less organic reagent, and simpler pretreatment procedure were integrated in this method. In sum, our developed method has good potential for simultaneous quantification of PZA, INH, and RMP in clinical practice, which may promote the effective clinical monitoring of TB.

## Data Availability

The raw data supporting the conclusion of this article will be made available by the authors, without undue reservation.

## Supplementary Material

Supplementary Table S1 Recovery of the analytes and IS in human plasma (*n*=5).

Supplementary Table S2 Intra-day (*n*=5) and Inter-day (*n*=15) precision and accuracy for 18 the analytes in human plasma.
