# Supplementary material for: A Simple and Sensitive UPLC–UV Method for Simultaneous Determination of Isoniazid, Pyrazinamide, and Rifampicin in Human Plasma and Its Application in Therapeutic Drug Monitoring
Source: Front Mol Biosci. 2022 Apr 29;9:873311. doi: 10.3389/fmolb.2022.873311 (PMC9099412; doi:10.3389/fmolb.2022.873311)
Supplement: Supplementary file 1 [file DataSheet1.pdf]

Supplementary Table 1 Recovery of the analytes and IS in human plasma (n=5)

	C(μg/mL)	Mean ±SD (%)	RSD(%)
INH	1	97.79 ±2.37	2.43
	5	92.08 ±4.41	4.79
	15	95.09 ±0.67	0.70
PZA	10	102.05 ±0.52	0.51
	20	86.61 ±0.49	0.56
	40	96.17 ±0.43	0.45
RMP	10	97.86 ±3.09	3.16
	20	97.08 ±1.67	1.72
	40	92.56 ±2.42	2.62
IS	30	90.85 ±2.20	2.43

Supplementary Table 2 Intra-day (n=5) and Inter-day (n=15) precision and accuracy for the analytes in human plasma

	C(μg/mL)	Intra-day (n=5)			Inter-day (n=15)		
		Mean ±SD	Accuracy%	RSD	Mean ±SD	Accuracy%	RSD
INH	1	0.966 ±0.04	96.56	4.58	0.975 ±0.04	97.45	4.09
	5	5.136 ±0.03	102.72	0.51	5.017 ±0.10	100.34	2.05
	15	15.205 ±0.02	101.36	0.14	15.125 ±0.07	100.83	0.45
PZA	10	10.009 ±0.01	100.09	0.11	9.756 ±0.22	97.56	2.30
	20	20.809 ±0.01	104.04	0.07	20.410 ±0.31	102.05	1.52
	40	40.605 ±0.05	101.51	0.13	40.377 ±0.19	100.94	0.48
RMP	10	9.001 ±0.16	90.11	1.74	8.816 ±0.34	88.16	3.82
	20	19.577 ±0.49	97.89	2.50	19.583 ±0.41	97.91	2.11
	40	41.876 ±1.56	104.69	3.73	41.982 ±1.14	104.96	2.72
